# A Health- and Resource-Oriented Perspective on NSLBP

**DOI:** 10.1155/2013/640690

**Published:** 2013-09-11

**Authors:** Cornelia Rolli Salathé, Achim Elfering

**Affiliations:** Department of Work and Organizational Psychology, Institute of Psychology, University of Bern, Fabrikstrasse 8, 3012 Bern, Switzerland

## Abstract

Nonspecific low back pain (NSLBP) is an important health issue of our time. Personal as well as economic factors, like suffering pain and experiencing disability on the one hand and enormous and still increasing costs to the economy and society on the other hand, display the importance of the matter. Tremendous research has been conducted in the last few decades on NSLBP. A PubMed search (June 17, 2013) on “low back pain” provided 22,980 hits, and when specifying for “low back pain, systematic review,” 3,134 hits were still generated. Most research has been done examining the development, risk factors, or therapeutic measures of NSLBP, but hardly any literature exists on resources related to NSLBP. The aims of this review are twofold. In order to shade light on the salutogenetic approach of NSLBP, and thus to focus on health instead of illness, the first aim is to facilitate the understanding of which therapeutic measures enhance the ability to cope with chronic NSLBP and enable (more) normal functioning in life. The second aim is to stimulate the understanding of resources protecting against the onset of NSLBP or against the development of chronic NSLBP and its resulting work absence.

## 1. Overview and Introduction to Nonspecific Low Back Pain

### 1.1. The Definition of Nonspecific Lumbar Back Pain

In order to examine resources of NSLBP, a definition of NSLBP is first given. NSLBP refers to pain symptoms anywhere in the lower back between the twelfth rib and the top of the legs [1]. It is defined as “pain or discomfort, localized below the costal margin and above the inferior gluteal folds, with or without leg pain” [2, page 171]. No recognizable, specific pathology such as infection, tumor, osteoporosis, fracture, radicular syndrome, or cauda equina syndrome is attributable to the pain sensations [3]. It further excludes organic referred pain. About four out of five persons experience low back pain at least once in their lifetime [4] with a one-year prevalence of 15% to 45% in industrialized countries [2]. Since the natural history of NSLBP is favorable, most individuals recover within six weeks [2, 5]. 

However, not all individuals recover spontaneously, and if NSLBP persists for longer than 12 weeks, acute NSLBP becomes chronic NSLBP [6]. An epidemiological study with data out of 16 European countries estimates that 19% of the European population suffered from chronic pain in 2003. The largest category—with 47% out of this 19%—is based upon back pain [7]. A recent inception study presents even higher numbers: more than 40% of 973 individuals developed chronic NSLBP after presenting themselves to primary care with acute NSLBP [8]. Chronic NSLBP differs from acute NSLBP in various aspects [9]. First are the chronological dimensions with acute NSLBP lasting less than four weeks, subacute NSLBP lingering for between four to twelve weeks, and chronic NSLBP persisting for longer than twelve weeks. Furthermore, sensory reasons also vary. Kröner-Herwig postulates that acute pain, for example, a knife wound or a sprain, is related to a distinct trigger, whose concrete function is to warn about an injury. Medical therapy aims to reach *restitutio ad integrum*, a complete healing. Psychological consequences include patients believing in healing and experiencing a locus of control [9]. Although this definition of acute pain is not completely transferable to NSLBP, since the pain triggers might remain unclear, the distinctions with chronic pain are noteworthy. The pain sensation of chronic NSLBP is no longer related to a possibly unclear peripheral trigger; pain is centralized [9]. Two kinds of neuronal plasticity, functional and structural, are relevant to this phenomenon [10]. Functional plasticity occurs rather quickly as a physiological adaptation measure. Neurotransmitters are distributed in different manners, while neuroreceptors change their receptor capacities. Structural plasticity relates to medium and long-term anatomical and biochemical modifications due to the altered requirements in the pain processing mechanisms [10]. For this reason, the chronic pain loses its function to warn. Factors, mechanisms, and treatment options of chronic NSLBP will be explained below. 

**Figure 1:**
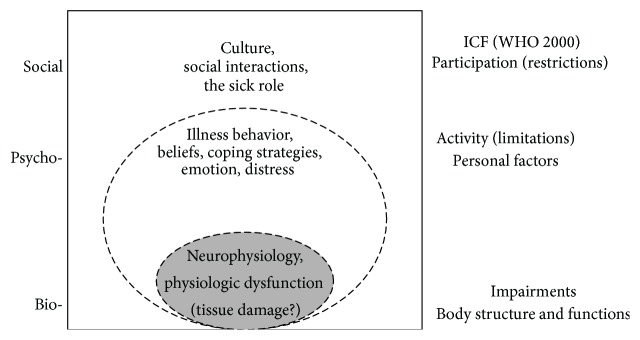
Biopsychosocial model of low back pain and disability [15, page 458].

Although pain symptoms are often implicitly attributed to medical reasons such as serious pathologies in the lumbar spine, other causes besides medical reasons also have to be considered as the origin. Therefore, NSLBP is often explained with the biopsychosocial model of pain [11, 12]. In his 1977 article, Engel postulated that the appearance of illness resulted from the interaction of diverse causal factors—biological, psychological, and social factors—and that psychosocial variables were crucial with respect to the susceptibility, severity, and course of illness. Engel also pointed out that the patient-clinician relationship influenced medical outcomes as well as scientific results with regard to the Hawthorne effect [13, 14]. (The Hawthorne effect relates to studies conducted in the twenties of the last century and refers to a noticeable change in the behavior of study participants without any experimental condition. The adaptations in the behaviors of the participants were explained by the arguments that participants knew they were observed and part of the study.) It was Waddell [15] who integrated the latest findings of the biopsychosocial model of NSLBP into the WHO-ICF model (Figure 1), the International Classification of Functioning, Disability, and Health, and took a further step toward establishing the model as a result.

A parallel research line toward an understanding of pain is Melzack and Wall's gate control theory of pain [16]. Two central aspects of the theory are crucial: the description of the transmission and modulation of nociceptive signals and the recognition of pain as a *psychophysiological phenomenon*. Much like Engel in 1959 [17, page 901], who mentioned the affective aspect of pain: “When we scrutinize more carefully the identifying quality of pain we note that it includes an affective tone. Pain is never neutral. It is usually unpleasant, but it may also be pleasant, if only in a relative sense. This effective quality brings pain into a very central position in terms of psychic development and function,” Melzack and Wall [16, page 978] stressed the psychological aspect by declaring that “the (gate control theory) model suggests that psychological factors such as past experience, attention, and emotion influence pain response and perception by acting on the gate control system.” 

Ever since then, a number of investigations have examined details of the gate control theory, as well as neurophysiological and neuroanatomical pathways of pain. Because its descriptions would clearly stretch this works' focus, interesting readers are referred to Butler and Moseley “Explain Pain” [18] for a broad overview, to Main and Colleagues “Pain Management” [19] for a detailed historical summary, or to Kröner-Herwig and Colleagues “Schmerzpsychotherapie” [20] for an overview. 

### 1.2. Development of Chronic NSLBP

**Figure 2:**
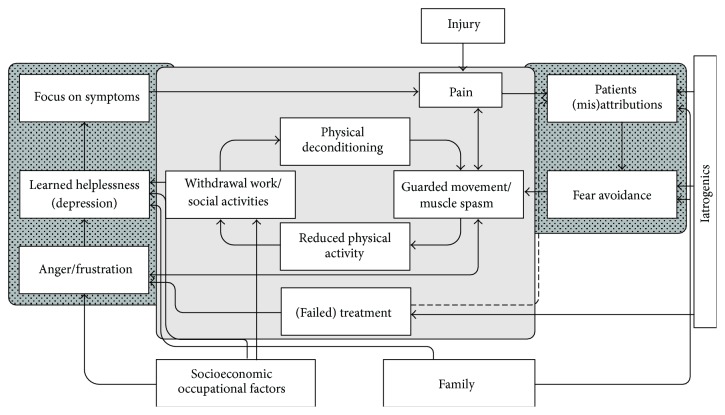
The modified Salford Model illustrating the development of disability [21, pages 97–104].

In the last decade, quite a few pain models have been published integrating interrelationships between complex factors which enhance chronic NSLBP. However, they often focus on *specific* pathways, such as psychological, biological, or behavioral risk factors of chronic NSLBP. By introducing the modified Salford Model [21], a broad overview of possible pathways to chronicity and of the momentary evidence-based knowledge is presented (Figure 2).

The Salford Model has been adapted in order to better demonstrate the impact of psychological factors (dark lateral squares) on the physiological dimensions (bright middle square). The arrows in the model indicate the directions of the relationships. A single arrow does not stand for a single transition; the model rather shows several, interrelating self-enhancing circles.

#### 1.2.1. Physiological Dimension

If an injury happens, nociception occurs, and the physiological reaction is pain. The model demonstrates now several *circuli virtuosi*, self-enhancing circles which can cause or increase chronicity. The basic circle is shown in the bright middle square. Pain often provokes guarded movements or muscle spasms [22, 23], which are reversible. Guarded movements lead to momentary reduced activity and decreased circles of actions, such as work or social activities. As a consequence, local physical deconditioning occurs by a decreased intra- and intermuscular coordination, as well as neuromuscular perception [23]. Until here, such a reaction can still be physiological. If, however, the circle mechanisms endure for too long and cannot be interrupted, the circle continues to increase the problem, and the reactions start to be pathological [23, 24]. 

#### 1.2.2. Impact of Psychological Factors

When turning toward the dark lateral square on the right, the self-enhancing circle of (mis)attributions and fear-avoidance behavior is pictured. Experiencing pain sensations, most individuals start to self-explain these sensations. Some might think not to worry or that the pain will pass in itself, while others start to worry about the nature of the pain sensation and search for signs of severe pathologies or false behavior. In particular if pain symptoms endure, misattributions and fear might increase [25]. Fear is a biological as well as a psychological response to an aversive stimulus. A normal coping behavior is to avoid such stimuli and restore homeostasis [26]. Beliefs and misattributions lead to a so-called fear-avoidance behavior in order to restore the homeostasis [27]. All movements which are subjectively prognosed to increase pain will be avoided, further guarded movements occur, and the basic circle keeps turning, as does the attribution circle.

The second dark lateral square on the left includes affective components of NSLBP. Prolonged guarded movements or muscle spasms can create or increase feelings of perceived injustice, thus anger or frustration [28]. Individuals start to become annoyed about the pain sensation and the resulting disability—which, again, can enhance muscle spasms or guarded movements as well. A normal and effective reaction to this anger is to ease the pain or to change the situation. If, however, all techniques and measures do not ease, and situations cannot be attained, feelings of helplessness or loss of control occur [29], which consequently can lead to psychological distress or even to depression [29]. Moreover, symptoms of depression and stress are found to mediate the effect of pain on disability [30, 31]. The emotional state of depressed individuals is altered, as they usually have a low mood that is accompanied by low self-esteem. Furthermore, they have often lost interest or pleasure in things that they used to enjoy [32]. Depressive individuals remember depressive experiences and thoughts more easily than past happy thoughts. The reason for this is the correspondence metaphor of memory. This declares that a similar state of emotion during learning eases the individuals' recall abilities and experiences. For example, learning something when happy will be harder to recall in a depressive state [33]. Therefore, the focus on symptoms remains and subsequently pain increases. Again, this circle keeps the basic circle turning. 

Further circles have to be introduced. First, when looking at the basic circle, withdrawal from work or social activities can also induce helplessness or psychological distress [34] and start or intensify the affective circle in this way. Second, iatrogenic influences need to be mentioned. Sometimes, treatment by medical staff is not successful [35]. Despite the willingness to help and heal, treatments can fail; thus symptoms, beliefs, or behavior become worse and can result in a failed treatment [36]. Unsurprizingly, this can lead to anger or frustration toward the medical staff [37], the hospital, or even the pain symptoms. Failed treatment can provoke learned helplessness, and support subjective (mis)attributions toward the symptoms [38]. Iatrogenic influences can also directly enhance (mis)attributions as well as fear-avoidance beliefs. Third, family or learnt behavior can have an impact on (mis)attributions, fear-avoidance beliefs, and behaviors [39], as well as learned helplessness [40]. Finally, socioeconomic and occupational factors, like high job stress levels [41], can cause withdrawal from work or social activities [42] and influence anger and frustration as well as learned helplessness [43]. Which is not shown in the figure is the fact that patients do not feel “understood” as a consequence and lose confidence, which leads to a bad doctor-patient relationship that also decreases treatment success [44]. 

Taken together, the Salford Model [21] gives an overview on how chronic NSLBP can develop and be maintained. Of particular note are two aspects. First, the basic circle in the bright square is physiological and only develops into a pathogenic pathway when it repeatedly occurs and cannot be interrupted. Second, psychological as well as exogenic processes can provoke a pathogenic development of NSLBP. However, the model has clear limitations. The complexity of the interrelations is shown to some part and one might assume how fragile the balance can sometimes be. Further endogenous factors, such as personal traits like introversion or neuroticism [45], self-efficacy [46], or resilience [47], as well as social factors like support from relatives or superiors at work [48] and material aspects [49] always influence possible confounding pathways. 

### 1.3. Risk Factors of NSLBP

With regard to the influencing factors of the development of (chronic) NSLBP, a clinical aim within the last 15 years has been to improve the understanding of risk factors which could serve in a prognostic way and help to identify individuals at risk developing prolonged NSLBP or transforming NSLBP to chronic LBP (e.g., [50–53]). As a result, flags containing different risk factors and obstacles to recovery have been developed [54–56]. The following paragraphs will introduce the established risk factors and flags. 

#### 1.3.1. Demographic Risk Factors for the Onset of NSLBP

The highest risk factor for experiencing NSLBP is a previous occurrence of NSLBP [57, 58], which is not surprizing considering the prevalence rate of NSLBP [4, 58]. As for body weight, a recent population-based study with more than 60,000 participants estimated a significant odds ratio (OR) per 5 kg/m^2^ increase in body mass index for men (OR = 1.07, 95% confidence interval: 1.03–1.12) and for women (OR = 1.17, 95% CI: 1.14–1.21) after controlling for age [59]. Other relevant characteristics are marital status, education, and employment. Being married, well-educated, and employed was negatively associated with costs due to NSLBP [60]. Again, specific inquiries reveal a slightly different perspective. A French population-based study on NSLBP and its risk factors illustrated a low educational level as crucial for the development of NSLBP. However, when considering work postures or heavy workloads, the educational level lost its predictability [61]. With regard to the underlying mechanisms, a low educational level and work postures have to be considered as confounders. The least-educated men demonstrated the highest frequency of physically tiring postures (63%), while the lowest frequencies of physically tiring postures were confirmed for the highest and second highest education levels [61]. 

Being female [62, 63] and being older than 50 years are well also defined risk factors [57, 63]. However, health behavior seems to differ with gender. While women demonstrate a higher probability of suffering of NSLBP and therefore to incur costs, men generate higher costs. The authors of the population-based German back pain study concluded that women tend to utilize healthcare more quickly than men, but that once men utilize healthcare it leads to higher costs on average [60]. 

Roughly summarized, there is evidence for demographic risk factors for the onset of NSLBP. However, evidence varies between studies and despite the investigations on prognostic factors, uncertainty remains regarding the strength of the associations and the extent of confounders [64]. 

#### 1.3.2. Risk Factors for the Transition from Acute to Chronic NSLBP

Risk factors mentioned in the flag system are often associated with a delayed recovery and thus with the transition from acute to chronic NSLBP [19, 54].

*Clinical Red Flags: Biomedical Factors*. Red flags are not considered as risk factors, rather as warning lights. Since NSLBP is defined as nonspecific, it is crucial to exclude possible medical problems. For that reason, red flags were first proposed by guidelines [65, 66] and applied in primary care to identify patients with an urgent need for a specialist opinion [19]. They were defined as medical-biomedical signs and symptoms that indicate an organic pathology or a concurrent serious medical problem. Examples are cauda equine syndrome signs, such as bladder or bowel incontinence, significant trauma, pain which gets worse when lying down, or unexpected weight loss with or without fever.

*Clinical Yellow Flags: Psychological/Behavioral Factors*. Yellow flags are defined as modifiable psychosocial or behavioral risk factors. They include subjective appraisals, unhelpful beliefs and expectations about pain, or negative expectations of recovery. These adverse cognitive appraisals can enhance the fear of movement, the avoidance of activities due to expectations of pain and possible reinjury, and feelings of being helpless, worried, and distressed [54, 67].

*Clinical Orange Flags: Psychiatric Symptoms*. Orange flags are defined as a mental health equivalent for red flags. They include excessively high levels of distress, severe personality disorders, drug and alcohol addiction, or clinical depression [19, 56].

*Occupational Blue Flags: Sociooccupational Factors*. Blue flags are defined as work-related risk factors. They include aspects of the employee and the workplace. With regard to the workplace, blue flags comprise physical job demands, low possibilities to modify work, or a stressful job environment. Likewise, regarding employee aspects, low job satisfaction, as well as low social support at work, can enhance delayed recovery [55, 68]. 

Two occupational constructs with strong evidence as risk factors for NSLBP shall be introduced more profoundly: (low) job satisfaction and (low) social support at work [69]. Job satisfaction includes an individual's thoughts and feelings about their work, with respect to the feeling of overall satisfaction or overall dissatisfaction [70]. Overall satisfaction can be understood not only as a single, global construct but also as an accumulation of various different facets like the work itself, social relationships at work, the supervisor, wages, or the travel distance to work [71]. Evidence about (global) job satisfaction and NSLBP is controversial. Low job satisfaction, in comparison to high job dissatisfaction, was found to act as a risk factor for NSLBP [69] or its transition to chronic NSLBP [72]. More recent literature did not confirm the influence of job satisfaction on prolonged sickness absence due to NSLBP [73, 74] or on the outcome of NSLBP in primary care settings [75]. However, satisfaction with one's job was described as a resource protecting against the development of chronic NSLBP and disability [72]. 

As for social support at work, various effects have been found for the promotion of health and healthy behavior [76]. Basically, social support implies that a person feels cared for and appreciated [77] and has access to help when needed [78, 79]. With respect to the complexity of social support, the literature differs between social support from colleagues or from superiors. Receiving support from close confidants had a detrimental effect on NSLBP, while support form supervisors or less confident colleagues correlated negatively with the NSLBP duration [48, 80]. These findings indicate that by focusing on neutral issues, support from more distant individuals does not disturb the integrity of the person seeking help [81]. (Or, in other words, although a more distant person supported an employee in need, the distance between the persons kept the supporting person from intervening too much. The employee in need did not succumb to a dependency towards the supporter.) 

*Occupational Black Flags: System and Contextual Factors*. Black flags are defined as the occupational and systemic context in which a person functions. They include misunderstandings or disagreements between key players, such as employers, or insurances due to financial and compensation problems. Further black flags include process delays with regard to treatment approval or financial security or social isolation due to dysfunction as a result of a lack of co-ordination among health care providers [55, 82]. 

### 1.4. Therapeutic Aims and Measures of Chronic NSLBP

Therapeutic aims include (a) the reduction of the factors responsible for pain maintenance and (b) the improvement of individual pain management [9]. With regard to the biopsychosocial aspect of NSLBP, the most highly recommended way of tackling chronic NSLBP is via a multidisciplinary treatment (MDT) program [6, 83]. MDTs are based on a cognitive-behavioral approach, which—ever since Turk and Colleagues integrated a cognitive-behavioral approach to pain [84]—has become the framework that most current pain management programs have been drawn from [26]. They focus on pain management rather than cure; contain a behavioral rather than disease perspective; enclose various measures like medical, manual, exercise, or psychological treatments; and include an interdisciplinary skill mix. The emphasis of the group therapy settings lies on active and self-helping approaches with regard to augmenting the patients' responsibility [26, 85]. 

### 1.5. Cost of NSLBP

Due to its high economic impact, the presentation of the economic perspective of NSLBP is important. Medical costs are divided into direct and indirect costs. Direct medical costs include physician consultations or medications, while direct, nonmedical costs incorporate transportation costs to attend medical appointments. Supplementary, indirect costs include decreased or lost productivity due to disability or sickness absence. Despite the difficulty in measuring indirect costs, it is well known that costs resulting from lost work productivity represent the majority of NSLBP-associated costs [86]. NSLBP costs estimations for Switzerland in 2005 were €2.6 billion for direct costs, representing 6.1% of the total healthcare expenditure. Indirect costs were estimated between €2.2 billion and €4.1 billion, depending on the economical approach or the individual productivity loss. The economical approach focuses on the time span until the productivity losses were compensated by a successor and assume lower productivity losses, whilst the overall individual productivity losses are summarized for the entire absence of a missing individual. The overall economic burden of NSLBP in 2005 was between 1.6 and 2.3% of the Swiss gross national product [87]. Most Western industrialized countries report comparable figures [86]. 

## 2. Work Absenteeism/Sickness Absence

Work absenteeism (WA), or sickness absence due to NSLBP, which quite often results in indirect costs mentioned above, also needs a brief overview. (Sickness absence is often used as a synonym to WA. However, the significance of the word might not be totally alike. Sickness absence refers to days absence due to a sickness—usually nonpermanent—while WA emphasizes the permanent absence from work due to a limiting factor, such as NSLBP.) Approximately 20% of the employees with a current NSLBP-episode experience long-term WA [88]. Nonspecific LBP is part of the problem causing WA, and it is well known that pain, disability, and WA are linked, but the relationship is complex and influenced by many factors (e.g., [89]). The influencing factors of WA share similar risk factor patterns to the influencing factors of NSLBP. For example, recovery expectations and fear-avoidance beliefs also belong to the psychosocial risk factors of WA [73, 90]. A recent study by Elfering and colleagues even proposed a relationship between the two factors. Fear-avoidance beliefs predicted the one-year recovery rate of NSLBP [91]. In addition, pain intensity [88], previous WA [92], and NSLBP disability or pain behavior [93, 94] were described as biomedical risk factors. Occupational risk factors comprised heavy physical workload [88] and low job control [92, 94]. High job insecurity, which could have been related to insecure organizational downsizing strategies [92], also belonged to the occupational risk factors. Finally, depression [94] or negative life events [92] further contributed to the development of WA. 

Clearly, WA is the most important impact of NSLBP. Its social as well as financial consequences explain the political interest in NSLBP and its possible resulting incapacity of work [89].

## 3. The Salutogenic Approach: A Health- and Resource-Oriented Perspective on NSLBP

Despite the amount of work studying NSLBP and its implications, a lot of questions remain unanswered about the mechanisms, confounding and risk factors, treatment measures, and the efficacy or cost effectiveness of those treatment measures for NSLBP [95]. With all of the impacts of NSLBP presented above in mind, a change of perspective through a new and resource-oriented approach towards NSLBP seems reasonable and warranted. More than twenty years ago, only a handful of studies had inquired about individuals who were considered NSLBP asymptomatic [96–100]. However, this specific line of inquiry was not of much interest in the 80s and 90s. Only recently has the scientific community seemed to recall the fact that approximately 20% of all individuals never experience NSLBP in their lifetime [101–103]. 

The emphasis in this review is on a health- and resource-oriented, hence *salutogenetic,* perspective of NSLBP. First, two quite approved salutogenetic approaches to NSLBP, the salutogenetic model and resilience, will be presented in order to broadly introduce the theoretical frameworks of resources. Second, the state of the art of health-enhancing resources will be introduced. Included in the state of the art are personal resources, behaviors, and physical resources, and occupational resources. The following discussion finally discusses all of the presented resources on the basis of the published technical literature. Practical implications as well as further outlooks complete this introduction. 

### 3.1. The Salutogenic Model

The salutogenic model, based on Antonovsky [104], focuses on resources enhancing recovery or keeping individuals healthy. Antonovsky [105] introduced the “sense of coherence” (SOC), which can be understood as a basic sense of trust or a stable feeling of confidence towards life. It includes three different components: comprehensibility, manageability, and meaningfulness. Thus, a high SOC proposes that internal as well as external stimuli are structured, predictable, and explainable (comprehensibility); that the individual has enough and eligible resources to cope with stressful situations (manageability); and that the external demands represent subjective challenges worth fighting for (meaningfulness) [106]. Antonovsky [105] further rejected the traditional dichotomous health versus illness model and postulated instead that the relationship between health and illness is dynamic and continuous. Therefore, he suggested a dynamic health ease/dis-ease continuum [107, page 15]. If, for example, a person cannot sufficiently cope with an external demand, a state of stress occurs, resulting in a change of the continuum towards the dis-ease end. However, by successfully coping with the stressor or resolving the state of stress, a change towards the ease and an increase in SOC arise. Previous studies have confirmed the relationship between SOC and general health [108], as well as LBP [109–111].

Although the salutogenic model is widely known, it is still controversially discussed. For example, SOC overlaps with other, well-established concepts like optimism [112, 113], self-efficacy [46], or locus of control [114], and the question remains whether SOC is a unique, clearly definable trait [115]. 

### 3.2. Resilience

A further salutogenetic concept is resilience or “psychological resistance.” It refers to one's capacity to navigate psychological, sociocultural, and physical resources that sustain well-being despite facing adverse and stressful situations and to provide these resources in daily routine [116]. For a broad overview see Fletcher and Sarkar [117]. Two different aspects of resilience are distinguished: resilience as a personal trait and relational resilience which includes the person-environment constellation [115]. 

If resilience is considered as a personal trait, the logic consequence investigating on resilience is to identify these resistant and resilient characteristics within individuals on certain outcomes. For example, Antonovsky's SOC [105] or Kobasa's Hardiness [118] is already established protective factors for negative impacts of stress. The latter, Hardiness, describes three core personality characteristics—to be in control, to show a high commitment, and to search for challenges. While these characteristics provide the motivation and courage needed to tackle a difficult situation, they also increase personal growth [119]. 

Alternatively, resilience can be described as a specific person-environmental constellation which varies over time. Protective factors may include personality characteristics like intelligence, personal aims, or coping strategies, as well as environmental characteristics including social network, educational style, or school support [120]. A crucial aspect is the individual's ability to develop and to change over their life and hence to successfully adapt to environmental situations [121]. This ability to adapt is the foundation of a stable and healthy personality [122]. 

Resilience is best understood as a resulting process of individuals interacting with their environments in order to endorse well-being or protect themselves against the influence of risk factors [47]. Therefore, a logical consequence is to ask for the processes and protective factors that endorse resilience by promoting well-being and protecting against risk [123, 124]. Currently, to the knowledge of the authors, no literature exists on resilience and NSLBP. However, two recent studies describe resilience as a new paradigm for adaptation to chronic pain [125, 126]. Though the main focus remains on individuals suffering from chronic pain. Nevertheless, processes and protective factors which protect against the onset of NSLBP have been examined and will be described in the next paragraph. 

### 3.3. Processes and Protective Factors against NSLBP

The following literature on processes and protective factors against NSLBP can be considered state of the art since no further literature was found in an extensive search for the period from January 1980 to July 2012 in the following electronic databases: Web of Science (ISI Web of Knowledge), MEDLINE (via PubMed), The Cochrane Library, PubMed Central, Ovid, and manually searched in Google Scholar with links to other articles taken from bibliographies. Selection criteria included personal, behavioral, physical, and occupational resources protecting against NSLBP, nonpregnant subjects above 18 years of age explicitly not suffering of NSLBP, and comparisons between samples with NSLBP or chronic NSLBP to healthy controls without acute NSLBP. The exception with regard to completeness, though, refers to physical resources. The presented physical resources give an overview of the current literature, but there remains no doubt about additional, undetected published literature describing physical differences between individuals with (chronic) NSLBP and healthy controls. 

However, before the resources protecting against NSLBP will be introduced, the handful of studies that have been inquiring about individuals who were considered NSLBP asymptomatic are briefly recollected and described.

#### 3.3.1. Studies on NSLBP-Asymptomatic Individuals

*Older Studies Inquiring NSLBP-Asymptomatic Patients*. Twenty years ago, five manuscripts dealt with the absence of NSLBP. All enquired about either physical and psychological working conditions [96, 99, 100], musculoskeletal status [96, 97], or life conditions [98] of individuals with an NSLBP-free lifetime prevalence. Two, possibly three of these studies are based on the same data [97, 99]. NSLBP-resilient participants (*n* = 36) who stated that they had never had NSLBP—or only occasionally very slight problems—and had never been sick-listed with NSLBP were recruited from a large manufactory. All statements were checked against the records of the social insurance office. Whether the participants of the third study, a previous pilot study, were related to the two studies by Hultman and colleagues [97, 99] is unclear, but this is a probability since 21 men within a similar age group were recruited from two enterprises. Again, statements in this third study were checked against records of the social insurance office [96]. In the fourth study, the Swedish Central Bureau of Statistics performed a cross-sectional study of NSLBP-resilient individuals (*n* = 1839) from a random geographically standardized 1 : 1000 sample of the Swedish population. They were asked by questionnaire if they had ever experienced any disease or illness related to NSLBP and whether they had suffered from NSLBP or sciatica [98]. No further reliability checks were performed for the answers. All participants were divided into three age groups. The prevalence of pain-free individuals was very high, with 72% in the youngest age group (30–39 years), 62% in the intermediate group (40–49 years), and 55% in the oldest group (50–59 years). Regarding methodological aspects, study three, the pilot study, assessed interview and physical examination data [96], and study two also assessed physical examination data [97], while studies one and four compared the questionnaire data of NSLBP-resilient persons with NSLBP sufferers [96, 99]. The fifth study dealt with construction workers. A total of 216 workers out of the 1773 questioned (12%) reported a “healthy lower back” without any history of NSLBP in a postal questionnaire. Prevalence rate decreased over time, and only 6% of workers reporting high stress levels were without back pain [100]. However, the reliability of the postal questionnaire selecting persons with healthy backs remains unclear.

*More Recent Studies Investigating NSLBP-Asymptomatic Individuals*. Two longitudinal studies assessed population-based, representative subgroups in Sweden and the United Kingdom. The Swedish study assessed a questionnaire at baseline with two followups of one and five years [103]. This was similar to the British study, which assessed followups at 15 months and four years after the baseline [101]. The Swedish study group around Reigo and colleagues [103] defined the absence of back problems as not having previous or ongoing NSLBP at any of the three evaluations and calculated adjustments based on the nonrespondent analysis at the baseline survey. They further examined two age groups, young adults (25–34 years), and older adults (54–59 years). Overall, 37% of the young and 43% of the older subgroup remained NSLBP-free. The British study asked their participants at each time point if they had suffered from any aches or pains which had lasted for one day or longer in the past month [101]. Persistent pain-free status was considered a measure of musculoskeletal health. Overall, 17.4% of the British study subgroup experienced no NSLBP. However, no study checked the pain-free reliability of the answers. 

A further study from Carragee and Cohen [102] observed NSLBP-asymptomatic soldiers who reported no NSLBP at the time of interview or in the previous three years. The authors discovered that 84% of all NSLBP-asymptomatic soldiers mentioned at least weak NSLBP at least once. Five percent of these soldiers suffered from disabilities. The surprizing fact was that five years after the initial data collection, 97% of all NSLBP asymptomatic soldiers still considered themselves as NSLBP-asymptomatic. Thus, Carragee and Cohen concluded that the subjective statements of NSLBP-asymptomatic individuals were not completely reliable and that an NSLBP recall bias existed. However, soldiers were assessed in a monthly interval at the end of each weekend drill [102]. When asked why they considered themselves NSLBP asymptomatic, the answers indicated that soldiers considered NSLBP after military drills not to be a medical problem—rather a “common fact of life”—or rather attributed NSLBP to the activities performed. For this reason only little or no medical care was sought [102]. Similar arguments were reported by Rolli Salathé and colleagues [127]. The 21 out of 42 NSLBP-resilient participants of the study who reported muscle tension in the back after sporting activities, a bad night, or gardening clearly defined these symptoms clearly as no pain. Overall, 42 NSLBP-resilient workers between 50 and 65 years of age were pairwise compared to propensity score-matched population-based case controls with and without momentary NSLBP. The aim of the study was to explore if the NSLBP resilient individuals had a better health, demonstrated more positive health behaviors, and were better able to achieve routine activities than the compared case control groups. Interestingly, the NSLBP resilient individuals differed from the controls without momentary LBP by being more vital, having a lower workload, a healthier attitude towards health, and by drinking less alcohol. The authors concluded that three underlying traits seemed to be relevant about NSLBP-resilient individuals: the personality, favourable work conditions, and subjective attitudes and attributions towards health [127]. 

#### 3.3.2. Personal Resources

The following paragraphs present results of the extensive literature search on personal resources. Cognitive appraisals and individual coping strategies are regarded as a distinct subgroup of personal resources, since relationships between personality, cognitive appraisals and coping strategies are well depicted [128]. 

Several personal resources, like the SOC, life satisfaction, or extraversion, have been described. For individuals without NSLBP, compared to individuals with chronic NSLBP, a higher level of SOC was mentioned in two studies. In the first study, the difference seemed to be influenced by stress manageability [129]. However, in the second study, the environment of persons with chronic NSLBP was perceived to be less comprehensible, manageable, and meaningful [130]. The same study found significantly higher levels of life satisfaction, extraversion, and less emotionality in individuals without NSLBP. In other studies, high life satisfaction prevented individuals with acute or subacute NSLBP from sickness absence due to NSLBP [131], while good mental health reduced the likelihood of persistent NSLBP in individuals with acute or subacute NSLBP after twelve-week followup [132].

#### 3.3.3. Cognitive Appraisals and Coping Strategies

The ability to seek, understand, and use health information is considered health literacy. A recent study examined broad elements of health literacy among individuals with no or chronic NSLBP. Out of the eight health literacy domains, only one domain was different between the two groups. Individuals without NSLBP scored higher in the domain “patient attitudes towards their health,” which included two personal abilities: first, the individual's ability to attend to the personal health needs; second, the individual's willingness to change or adapt their personal lifestyle to maintain their health state [133]. The authors concluded that individuals with chronic NSLBP seem to have greater difficulty engaging in general proactive health behaviors. In a different study, a large sample of NSLBP-free individuals was prospectively examined over four years. In a univariate analysis, individual characteristics included low anxiety and low health anxiety, as well as low depression and only a few recent adverse life events [101], indicating that individuals without LBP seem to be emotionally stable and do not often have to cope with stressful life events. 

#### 3.3.4. Behaviors

*Healthy Behaviors*. Two different health behaviors have been found in the extensive literature search regarding personal behaviors. For this reason, the first paragraph will illustrate healthy behaviors, whereas the second paragraph highlights risky health behaviors. 

With regard to Briggs' conclusion [133], general proactive health behaviors, such as taking part in sports and being active, seem to influence NSLBP in a positive way. By exploring salutogenetic factors of chronic LBP, participating in sports was found to decrease the degree of chronic NSLBP [134]. Furthermore, the influence of subjective workload on the degree of NSLBP was moderated by sports activity. For individuals doing sports more than twice per week, subjective work load no longer enhanced NSLBP [134]. Similar results confirmed these findings: for example, individuals without NSLBP were more often moderately physically active for one to two hours during their leisure time, [135], as well as more regularly and more enduring physically active than NSLBP-sufferers [136]. Further, they were better able to do routine activities such as climbing stairs or regular walking [127]. Although one study found better health status to be associated with lower medical care, Saraste and Hultman [98] could not confirm differences in activity and leisure time behaviors. However, a recent study illustrated that individuals without NSLBP walked 0.7 hours longer per day, accomplished 3480 steps more per day, and had an altered physical activity pattern than individuals with chronic NSLBP [137]. 

Sleep as a means to reload strength and energy is considered a health behavior. Sleep behavior appears to be different in individuals with or without NSLBP. While sleep duration does not play an important role in the differentiation, sleep quality does [101, 138]. Subjective sleep quality can be divided into self-reported sleep onset latency and self-reported sleep efficacy. On the other hand, objective sleep quality is divided into sleep efficacy and waking after sleep onset, both of which can be measured using actigraphy. Individuals without NSLBP scored better in both domains than individuals with chronic NSLBP [139]. Furthermore, the study identified significant associations between NSLBP, physical health, and disability levels, as well as the subjective, but not objective, sleep quality in the group with chronic NSLBP. 

*Risky Health Behaviors*. Referring to risky health behaviors, like smoking or drinking alcohol, to the knowledge of the authors only two studies found higher alcohol consumption for individuals with NSLBP versus healthy individuals. NSLBP-resilient individuals drank significantly less alcohol than individuals without momentary NSLBP but did not differ from individuals with momentary NSLBP [127]. However, the results of the second study are based on a univariate analysis and could not be confirmed by the multivariate analysis [140]. Besides, individuals without NSLBP appear to be less frequent smokers [98, 135, 136, 141] although the gender question is not yet exclusively answered. Björck-van Dijken and colleagues [135] found evidence for more frequent NSLBP-free female nonsmokers, while Saraste and Hultman [98] described this phenomenon for 50–59 year-old males only. Moreover, smoking seems to affect the extensor muscle strength: non-smokers without NSLBP seem to have stronger back muscles than non-smokers with NSLBP, while no distinction could be found between the smokers [142]. 

Before turning to the physical resources, a short summary of the personal and behavioral resources will be given. Individuals without NSLBP appear to have more personal resources, like higher levels of SOC and life satisfaction and less emotional instability, and were more able to attend to personal health needs. With regard to health behaviors, individuals without NSLBP were more physically active and had a better subjective as well as objective sleep quality, whereas risky health behaviors, like smoking or drinking alcohol, were less common. 

#### 3.3.5. Physical Resources

Although physical resources are the most well-investigated resource factors, they have not been included in the extensive literature search. However, it is common to compare two subgroups in medical literature in order to differentiate between individuals with and without NSLBP symptoms. Therefore, all of the findings described in the following section refer to such investigations. Examinations have been performed on six, highly interrelated aspects: (a) higher order kinematics during complex movement tasks, such as displacement, velocity, or acceleration; (b) proprioception of the spine; (c) spinal movement patterns; (d) postural control; (e) body perception; and (f) muscle strength.

*Higher Order Kinematics and Proprioception of the Spine*. Higher order kinematics in complex movements seem to distinguish well between NSLBP-sufferers and healthy individuals [143]. The neurophysiological foundation of higher order kinematics, however, is the proprioception of the spine (b). This is described as “[…] the sense of position and movement of one's own limbs and body without using vision. There are two submodalities of proprioception: the sense of the stationary position of the limbs (limb-position sense) and the sense of limb movement (kinesthesia)” [144, page 443]. Investigating proprioceptive aspects, studies have asked participants to reproduce predetermined target body positions, such as standing or four-point kneeling [145, 146]. If participants did not manage to achieve the position, the repositioning error was calculated as the absolute difference between the target position and the participant-perceived target position [147]. All three studies found significant, but not the same, differences in lumbar proprioception between individuals with and without NSLBP. Newcomer and colleagues [147] described controversy repositioning errors for flexion and extension movements with a higher repositioning error for flexion and a lower error for extension in individuals with NSLBP. In addition, Descarreaux and colleagues [146] found modifications in movement time, peak velocity, and acceleration in some, but not all, NSLBP-study participants. No significant differences in repositioning tasks were reported by Lee and colleagues. However, they found a greater motion perception threshold in NSLBP patients than in healthy individuals [148]. A similar result was reported regarding the pre- and postlumbosacral position sense after paraspinal muscle vibration. Individuals with NSLBP had a less refined position sense due to altered paraspinal muscle spindle afferences and central processing of sensory input [149].

*Spinal Movement Patterns and Postural Control*. With reference to spinal movement patterns (c) and postural control (d), lumbar and hip movements were investigated before and in response to rapid bilateral arm flexion movements while participants were asked to control their trunk in motion. Individuals with NSLBP used the preparatory extension of the lumbar spine less frequently and provoked a greater spinal displacement, which was induced by shoulder flexion [150]. A further aspect of postural control is body sway, which is defined as deviation of the body away from the center of the body's gravitation line. Multiple factors are attributed to causing body sway, for example, an “inherent noise within the human neuromotor system, (or a) reflexive of an active anticipatory search process, or an output of a control process to maintain postural control” [151, page 358]. Two recent reviews illustrated the association with an increase in anteroposterior body sway in individuals with NSLBP exhibiting a greater postural instability than healthy individuals [151, 152]. Similar results were described in a study exploring the balance performance in unstable sitting: individuals with NSLBP showed poorer balance performances and delayed lumbar muscle response times in the highest difficult balance task levels, compared to healthy individuals [153]. In order to find possible mechanisms of postural control strategies, a study investigated the body sway of individuals with and without NSLBP by manipulating the acute inspiratory muscles fatigue (IMF) [154]. After IMF, individuals without—much alike the individuals with—NSLBP used a more rigid proprioceptive control strategy instead of the normal multisegmental control, as in a nonfatigue condition. This resulted in decreased postural stability [154].

*Body Perception and Muscle Strength*. Regarding body awareness or body perception (e), some recent studies have been exploring the body image, body schema, or the tactile acuity by testing the two-point discrimination on the back. Comparing the accuracy of trunk rotation judgment in individuals with bilateral or monolateral NSLBP versus individuals without NSLBP, a decrease in the accuracy was found for each group. Healthy individuals achieved a 20%, respective to 33%, higher accuracy than individuals suffering from monolateral, respective to bilateral, NSLBP [155]. In addition, a decreased tactile acuity was found in the area of usual pain in individuals with chronic NSLBP, indicating a distorted body image [156]. Likewise, a larger two point discrimination threshold was found in persons with NSLBP [157]. Tactile acuity is described as a clear signature of primary sensory cortex organization [157]; therefore decreased tactile acuity might refer to a change in primary sensory cortex organization. A recent review portrayed such functional as well as structural brain changes in chronic NSLBP [158]. A change in cortical representation resulted in changed cortical activity and responsiveness, with implications for the response pattern to noxious stimuli, psychological and cognitive effects, and altered body perceptions [158]. 

As for Hultman [96], who revealed flexible backs, flexible hamstrings muscles, and stronger extensor compared to flexor isometric muscle strength, a prospective study over five years with 67 NSLBP-healthy persons described the extensor/flexor muscle strength ratio as the most sensitive parameter for the onset of NSLBP. Higher extensor than flexor muscle strength appears to be a resource preventing NSLBP [159], but the literature reports inconsistent findings regarding the association between NSLBP and trunk muscle function [160]. However, individuals with NSLBP were reported to have a pelvic floor muscle dysfunction compared to healthy individuals [161]. In addition, a systematic review on prospective high quality controlled trials examined clinical interventions to prevent self-reported NSLBP in working-age adults. The only treatment found to be effective was exercise. The aims of exercise mentioned in the included studies were to increase muscular strength, endurance, flexibility, and postural control [162]. Regarding the spinal muscle population, a systemic review on medical imaging studies revealed a paraspinal muscle wasting with reductions in fiber density, fiber atrophy, and fiber conversion from Type I (slow-twitch fibers) to Type II (fast-twitch fibers) in individuals with chronic NSLBP. Meanwhile, in back healthy individuals, paraspinal muscles contained a high proportion of Type I fibers, which played a crucial role in maintaining posture [163]. It appears that the results from Hultman and colleagues [97], describing thicker and more enduring back muscles, have influenced the formation of the systematic review by Demoulin and colleagues [163]. 

Taken together, individuals without NSLBP have many physical resources such as a better proprioception of the spine and overall better postural control. In addition, body perception and muscle strength seem to be different in individuals without NSLBP. 

#### 3.3.6. Occupational Resources

*Physical Work Resources*. The absence of a physically heavy workload in relation to individuals without NSLBP has been described in various studies [69, 98–100, 127, 140, 164]. However, when prospectively comparing two different age groups over five years, only the older workers without NSLBP (aged 54–59 years) experienced a significantly lower physically heavy workload [103]. Nevertheless, this finding has been recently confirmed in a younger prospective subgroup as well: high physical workload was identified as the greatest risk factor for the onset of NSLBP in 2,235 newly educated female health care workers without prior NSLBP history, one and two years after graduation [165]. Further physical work resources, like an appropriate work posture or the absence of bent body positions, were quite often investigated by comparing individuals with and without NSLBP (e.g., [99, 166]). When considering the duration of aversive postures at work, a period of less than two hours a day or the ability to change posture seems to prevent the onset of NSLBP [167].

*Psychological Work Resources*. The effects of social support were also described from a salutogenetic point of view. Individuals without NSLBP perceived a high degree of freedom [96] and higher levels of social support [130]. Additionally, social support was found to prevent the development from acute or subacute to chronic NSLBP [132]. Moreover, two studies presented moderation effects of social support. In the first study, social support moderated the degree of chronic NSLBP in that individuals with a very high subjective work load and high social support experienced significantly less chronic NSLBP than individuals with low social support [134]. The second study revealed that high social support buffered sickness absence at baseline as well as the impact of sickness absence one year later. Thus, individuals with high sickness absence at baseline and high social support were no more absent from work after one year than the group with low baseline absence. However, the impact of high baseline sickness absence on individuals with poor social support resulted in high sickness absence after one year [131]. Similar findings were reported for high job satisfaction. Individuals with a high to very high subjective work load and job satisfaction above the median level experienced less chronic NSLBP than individuals with job satisfaction below the median level [96, 134], while high levels of job satisfaction buffered sickness absence at baseline as well as the impact of high sickness absence one year later [131]. However, results for job satisfaction were controversial. First, the correlation between higher levels of job satisfaction and the absence of NSLBP was only found in older workers when prospectively compared to a younger working group [103]. Second, high job satisfaction was unexpectedly positively correlated to NSLBP [164]. Further work is needed to explore the relationship between NSLBP and job satisfaction. 

The absence of psychological distress seems to be a resource for the absence of NSLBP as well [101, 141]. However, psychological distress can be understood in broader associations like socioeconomic or psychologically demanding situations too. A high income category or good qualifications were resources for protecting against NSLBP [140, 168]. Similar findings were portrayed for the absence of psychologically demanding situations, like the inconsistency between job and educational level or excessive demands in the workplace [99, 164]. In line with these thoughts, pain-related fear or fear avoidance can also enhance psychological distress. Thus, the absence of fear-avoidance beliefs or pain-related fear had a predictive effect on the absence of NSLBP after one year [90, 167]. 

Finally, the absence of a stressful job was mentioned as a resource against the onset of NSLBP, although it was only found for the younger workers (aged 25–34 years) and not for the older subgroup [103]. Again in line with a stressful job, the absence of a hectic work tempo or a blue collar job was revealed to be resources against the nonacute NSLBP [140]. 

Taken together, personal, physical, and occupational resources that protect against the onset of NSLBP or the transition from acute to chronic NSLBP exist. Back healthy individuals seem to be healthier overall, both physically and psychologically; they appear to engage more often in proactive health behavior, demonstrate a higher sleep quality, and perform less risky health behaviors like smoking or drinking alcohol. The general “positivity” is recapitulated within the available physical resources like a good proprioception of the spine, good postural control, a better body perception, and higher muscle strength. Also, there is absence of a physically heavy workload and awry body positions at work as well as the surplus of psychological work resources such as social support, high job control, or the absence of psychological distress.

### 3.4. Conclusion and Further Prospects

Acute as well as chronic NSLBP is a prominent and highly relevant personal and economic problem of our time. Although the last decade has been the “Bone and Joint Decade 2000–2010” [169] provoking a tremendous amount of research, NSLBP remains a book with seven seals. A lot of questions still need to be answered. By taking a different point of view, the salutogenetic aspect of NSLBP, the problem is tackled nonpathologically with the aim of looking for positive, health-enhancing perspectives. 

## 4. Discussion

With regard to a resource-oriented approach to NSLBP, some findings need to be further discussed. The comparison of a NSLBP-resilient group to a case control group with momentary LBP [127] confirmed previous findings. An univariate analysis identified for the NSLBP group a better overall health state [98], fewer musculoskeletal as well as overall comorbidities [170, 171], a higher life satisfaction [130], a higher sleep quality [101, 138, 139], a better appreciation of their own health [133], and easier routine activities for LBP-resilient individuals [134, 135, 137]. However, with regard to vitality, the most prominent health factor differing between NSLBP-resilient individuals and both control groups [127], no previous literature was found in the salutogenetic perspective. Nevertheless, two recent studies included low vitality to predict a poor outcome for NSLBP patients. The first study examined a sample of NSLBP patients in primary care [172]. The second study created distinct comorbidity clusters which predicted the NSLBP or neck/shoulder pain diagnosis probability in an adolescent sample from Australia [173]. The clusters defined by the latter study included a “healthy individuals' cluster” with a low probability of the diagnosis NSLBP or any other medical condition. Not only did vitality differ in a highly significant manner from the other three clusters, but Beales and colleagues [173] also related the groups' resilience towards positive beliefs and appreciations about health. Further investigations might validate the creation of four distinct clusters and might further examine prospective developments of NSLBP resilience.

When focusing on the case control group without momentary NSLBP, compared to the NSLBP-resilient group, some differences were still detected [127]. Univariate analysis revealed a higher vitality, greater personal importance of exercise, and lower alcohol consumption for the NSLBP-resilient group. Multivariate analysis further added significantly lower workload. Before discussing workload, exercise and its implication towards NSLBP will be briefly looked at. Although the importance of physical activity with regard to NSLBP prevention is often implicitly assumed, scientific evidence is controversial. One systematic review clearly confirms this assumption [162], while another recent systematic review pointed out that intense physical exertion during leisure time was moderately associated with NSLBP and that everyday physical activities prevented the onset of NSLBP [174]. The answer to physical activity as a resource for NSLBP might be its frequency [175]. 

With all of the evidence identified, the prognostic relevance for a low workload is beyond doubt [69, 99, 127, 140]. However, this significant segregation supplies an even heavier argument: workload even differs between LBP-resilient individuals and those without momentary NSLBP—of whom we do not know if and how often they have suffered from NSLBP before. Clearly, employers, and employees as well, carry a tremendous responsibility with regard to prevention measures by providing adequate and safe workplaces with high quality tools. It is, however, the responsibility of every individual to use the tools provided and to implement the health guidelines relating to lifting activities.

Life satisfaction was hardly mentioned as a resource variable before [131]. Rather, dissatisfaction with life was found to predict an NSLBP incidence within a year [176]. When compared to individuals without chronic disease, individuals with chronic NSLBP demonstrated less life satisfaction [130], yet life satisfaction seemed to be relatively stable for individuals with chronic NSLBP despite different treatment measures. Even though it slightly improved over the course of four years, the baseline and follow-up scores of life satisfaction were very much alike between individuals treated with a lumbar fusion versus conservative cognitive interventions and exercise [177]. However, life satisfaction is modifiable. Working on acceptance strategies with the aim of increasing individuals' ability to behave according to interfering pain and distress, life satisfaction improved significantly over the course of seven months [178]. 

### 4.1. Further Prospects and Implications

After grappling so intensively with resources of NSLBP, one might wonder why? It is certainly true that something affirmative like a resource is principally positive. However, this is not sufficient. Resources can moderate the dealing with pain, illness, or disability. This might influence not only the treated persons' perspective, but the treating persons' perspective as well. First, the patients' prospect will be discussed. A physical limitation like NSLBP, but even more, chronic NSLBP, can change individuals' life. “Normal activities” are forced into the background by upcoming limitations or disabilities due to NSLBP and pain enters to the center of attention. In such a case, an individual might start feeling disabled, worthless, or lost in pain. This development might even be aggravated in individuals with a strong dichotomous attitude; for example, being healthy and able to work is good, while being ill and disabled is bad [179]. Resources might relieve this vicious circle of chronic NSLBP by offering new perspectives. An individual might detect competences, skills, or abilities despite chronic NSLBP. One might have learned to be worthy, recognized, and able to work despite the physical limitations. In short, resources might add a more sophisticated view of a persons' life and of how to stay active despite pain. McCracken and colleagues, who investigated psychological acceptance of chronic pain, speak about “psychological flexibility” which may reduce the impact of chronic pain [180]. This psychological flexibility not only is significant for the individual but also has implications for economic and health-political aspects, for example, work organizations as well as invalidity insurances.

From the other point of view, the physician's or clinical specialist's side, working every day with individuals in pain might not be easy psychologically. Treating persons in a health practitioner's clinic, for example, might generate an individual strategic medical management that relies on tacit knowledge rather than on guidelines [181, 182]. This individual strategic medical management might include a “feeling” about who will pursue an easier healing process and with whom the situation might get difficult [183]. By examining typical clinical situations that focus on preventing either the transition from acute to chronic NSLBP or prolonged sickness absence, results might underline, confirm, or even supplement clinical tacit knowledge. Since pain, disability, grief, or general (health) problems dominate the daily clinical business, a change from totally pathological towards a resource-added perspective might enhance work quality aspects of physicians or clinical specialists, might further simplify the physician/clinical specialist-patient relationship, and might finally empower patients pain self-efficacy [184]. Further research should investigate into resource-added treatments and possible outcomes as well as the implications for patients and treating persons with regard to work quality aspects. 

An international agreement has been reached for therapeutic aims and treatment measures for chronic NSLBP [185]. This includes a discouraged use of passive treatments like modalities, medication, or manipulation and motivates a focus on active measures like supervised exercise therapy, cognitive-behavioral therapy, or multidisciplinary treatments. However, two aspects should be pointed out. First, not all persons with chronic NSLBP are thus treated [186]; second, with only moderate efficacy and not everybody benefiting from standardized treatments, trends tend to move in the direction of using different therapeutic measures for different patient subgroups [187, 188]. Nevertheless, different therapeutic measures for different patient subgroups do not call into question the acquisition of resources. To retrieve resources as well as to enlarge the perception of pleasant life aspects should belong to an interdisciplinary, standardized, cognitive-behavioral based program (e.g., [189]). Above and beyond, with regard to a salutogenetic approach to NSLBP, some questions need to be asked. Why should primary care staff like physicians, clinical specialists, care assistants—or indeed medical specialists—not benefit from experiences gained and learn from the knowledge acquired in interdisciplinary, specialized pain clinics? Should primary care treatment measures only address risk factors of NSLBP and relating coping strategies? Why not peek towards a health- and resource-oriented perspective and include health-promoting measures in primary care? Focusing on resources despite NSLBP might improve the patient-physician/therapist relationship and might moderate not only the therapeutic outcome (e.g., [190]) but also the NSLBP patients' therapeutic benefit, which is shown in the patient satisfaction [191]. Further research should address the moderating effects of resources, such as sensory perception, moderate activity, or social as well as therapeutic relationships, on function instead of impairment.

Associations with a theme such as NSLBP always reveal medical considerations first. However, a biopsychosocial problem has to influence other aspects as well. Consequently, NSLBP also affects working environment. Increased knowledge on resource factors may enhance preventive behavior in personal and occupational settings, minimize work absenteeism, and decrease socioeconomic costs. In a more detailed view, a person being absent from work due to NSLBP might decrease productivity or reduce available expertise in a working team. Enhanced stress such as time pressures or isolation due to fewer interactions between workers, increased concerns, or difficult supervisor-employee relationships might arise in a team. How could resources influence such difficult work situations? One side of the answer includes the employee (or patient) view already mentioned above. Resources might add a more sophisticated view of a persons' life and of how to stay active despite pain. Resources might even keep the individual in work. This would absolutely correspond to the fifth revision of the Swiss Disability Insurance (fifth IV revision). A main objective of this revision is to integrate individuals at risk of disability and work absenteeism early. However, resources might tackle the problem from the other end. 

With regard to the employers' side, the same question as mentioned above arises: Why do employers or supervisors not benefit from experiences gained and learn from knowledge acquired in interdisciplinary, specialized pain clinics? One answer might be because employers do not have a therapeutic mission. Yet, regarding the fifth revision of the Swiss Disability Insurance, it is not clear at all if employers are not told to take responsibility for their employees' health and work ability. Nevertheless, resources factors like social support at work or job satisfaction might not require vast investigations in new technology or better-quality material. Simple things like a better supervisor-employee relationship (e.g., [192]), appreciation and valorization for the work done [193], or even a little financial recognition might improve job satisfaction [194]. Further research will have to prove these statements. 

The last point to mention is that scarce literature that deals with NSLBP-asymptomatic individuals [101–103, 127] exists. Most of what is known involves so-called healthy controls. Individuals experiencing no acute NSLBP are compared as a control group to individuals with acute, subacute, intermittent, or chronic NSLBP. Unfortunately, the control group never achieved grand popularity nor standardized definitions. A NSLBP-healthy control group is a group of individuals without acute NSLBP, yet nothing is known about the prior incidence or recurrence of NSLBP. In order to facilitate the distinction between NSLBP-asymptomatic individuals and individuals without current NSLBP, the scientific community ought to realize first that differences exist. This requires more investigations with the intention to gain new insight about psychological, behavioral, physical, occupational, and even neurological characteristics of NSLBP-resilient individuals in specific person-environment constellations. Findings in such inquires might be included not only in medical and therapeutical, but also in pedagogial, as well as occupational settings in order to enhance NSLBP resilience. However, before such specific person-environment constellations can be proven efficiently, there is still plenty of work ahead. Also, one might not forget that the study sample will not be easy to collect since NSLBP-resilient individuals are rather difficult to locate, especially in the age group of above 50 years [57]. 

## 5. Conclusion

Nonspecific low back pain is a dominant problem of our time with severe personal as well as occupational restrictions. The existence of health-promoting resources should be introduced gradually to the attention of the scientific community as well as the clinical staff at the patient front line and supervisors in their daily work although perspectives are often back breaking.
